# Poetry, Music, and Health

**Published:** 1878-02

**Authors:** 


### HALL'S JOURNAL OF HEALTH.

Our Legitimate Scope is almost boundless: for whatever begets pleasurable and harmless feelings, promotes Health; and whatever induces disagreeable sensations, engenders Disease.

WE AIM TO SHOW HOW DISEASE MAY BE AVOIDED, AND THAT IT IS BEST, WHEN SICKNESS COMES, TO TAKE NO MEDICINE WITHOUT CONSULTING A PHYSICIAN.

Vol. 25.] FEBRUARY, 1878. [No. 2.

### POETRY, MUSIC, AND HEALTH.

MANY persons, when hungry, are so "ugly" and irritable, that they remind us of a parcel of starving pigs called up to the slop-trough of a farmer's kitchen; they will grunt and push and squeal and bite one another with surprising vigor, until they get to eating fairly, when there is a sudden and all-pervading silence, with scarcely any evidence of life, except the wagging of their tails, in token of profound satisfaction with the[illegible]lves and all the world; when perfectly filled, they retire in d[illegible]ified silence, and take their siesta on the sunny side of some fence or wall, in the most benignant humor imaginable. Children who are hungry, often come to the table in the same mood; and discreditable as the announcement may seem, many parents, not unpossessed of some excellent traits of character, exhibit, on their entrance into the dining-room, such a fretful and complaining nature, that any inquiry, however kind, courteous, or conciliating, is almost sure to be met with an insulting silence, an impatient reply, or a downright boorish rejoinder, showing very conclusively, that in temper, in disposition, and nature, they are not much above "the brutes which perish." Many a notable affectionate and loving-hearted wife, after exercising all her ingenuity in preparing an inviting meal for her husband, often waits patiently, and yet vainly, for some expression which recognizes her fidelity to household duties; others more unfortunate still, have no reward but querulousness and ungracious fault-finding. When the meal is over, these "monster" husbands return to their "right mind," and are every whit as gracious and good-natured as any other pigs.

There are some who are subject at periods to an ugliness of disposition, which excites a conjecture that possibly they may be "possessed of a devil," sometimes two or three or more—transiently, at least; others there are, beyond all question, who have always had that companionship; and forty thousand woes be to the unfortunate individual who has such a yoke-fellow—*the devil of habitual ill-nature*, beginning with the early morning, ceasing only with the exhaustion which gives sleep.

There was known to be a cure for the acute form of this malady, three thousand years ago, for it was said of a certain king, that he was subject to these "spells" of devilishness; and that on one occasion, the evil spirit left him, and he "was well," as soon as the skillful and handsome son of Jesse took down his harp and swept its strings with the fingers of an amateur. Whether there was an accompaniment of "thoughts that breathe and words that burn," is not certainly known, but as David has written some of the sweetest, and some of the sublimest poetry which has fallen from the pen of mortals, it is not impossible that he sang when he played; and the result certainly was, that whether it was music or recitation, or both, the evil spirit was put to flight, and the royal patient was pronounced "well," without the necessity of a strait-jacket, pills, castor-oil, or chloroform.

It is the fashion of the times, however, to take it for granted, that this evil spirit, whose origin is from below, the spirit of fretfulness, of dissatisfaction, of incessant fault-finding, and chronic ill-nature, as exhibited in domestic life, can by no possibility exist on the diviner side of the house; but, as a matter of course, can only be found in the lords of creation; hence, or for other reasons, every mother in the land is at more pains, and has more solicitude for her daughters' musical training, than for any thing else, as if it were to be expected, as a matter of course, that all husbands had to be exorcised. And it is a fact, that if any man had forty thousand Beelzebubs tearing round within him, making a very Pandemonium in the housenold, every individual one would scamper off with the rapidity attributed in olden time to a shot placed in particular circumstances on a shovel, the very instant that Beauty's voice swelled the notes, and tapered fingers swept the octaves.

While, therefore, it is philosophical to have our daughters learn music, it might be well to remember that "spirits differ." Some men have no ear for music; have no music in their souls, while all have more or less of human nature; more or less of the leaven of ill-temper, of impatience and wrathfulness, which is not amenable to the symphony of sweet sounds, but which is softened down to the lovingness of a baby's cooing at the exhibition of a little common-sense; of tidiness of person; of worldly prudence; of domestic management and household handiness on the part of the wife. No man possessed of any force of character can bear with equanimity the daily observation of the fact, that what he brings into the house for the comfort and sustenance of his family is not taken care of, is destroyed by unprincipled servants, or used with a criminal lavishness which benefits nobody, and yet is an hourly injury to him, inasmuch as the fruit of his labor and his care is ruinously used.

The demon of deep dissatisfaction will take possession of the man who has any respect for himself, his family, and his social position, when he begins to find out that his wife "has no taste for housekeeping;" that this branch of domestic duty is left entirely to the servants, and, as a consequence, the carpets are moth-eaten the first summer; the costly furniture in six months looks as if it had been in use a dozen years; the rosewood is "nicked;" the sienite marble is stained with all the colors not belonging to it; the costliest velvets and tapestries are irremediably greased; while the walls are scratched and match-marked in every possible direction.

It can not be a just matter of surprise, that a man should become possessed of an evil spirit, when he finds that as often as he presents his wife with a charming "hat," with a splendid "silk," with a magnificent set of furs, he is doomed in less than a week to find the "love of a bonnet" lying about, first on a bed, next on a center-table, next hitched on to the hatrack in the hall, as if it were a mere "hack," to be put on only when it was like to rain, or when going out to make "next door" a neighborly visit after nightfall; or if the costly silk, after the first wearing, has been hastily dumped down on the floor, or hurriedly crammed into a drawer, to be taken out with a hundred thousand unsightly creases; or if the diamond breastpin is broken, or the bracelet-guard lost, or a diamond is missing from the finger-ring after the first wearing. Not a less powerful means of "bringing up" an evil spirit into a man, is the finding his house all topsy-turvy when he comes home after the business of the day; the children crying, the servants "in a stew," while the wife is in a humor so ungracious, that the moment her husband enters the door, she begins with the volubility of a dozen ordinary women, to pour out one complaint after another, about every servant and every child; about the butcher and the baker and the milkman, ending with an intimation of a very unmistakable character: "It's your fault." And if, after all this, the five o'clock dinner is placed on the table at six, the potatoes hard, the roast beef black, the bread half dough, the milk sour, and the soup dishwatery, it can not be surprising, if evil spirits do "catch him" up and "whisk" him off to the village-tavern, the grog-shop, the billiard-saloon, or the gaming-table, returning home later and later, until, after a while, he habitually enters his house in the small hours of the morning, beastly drunk, and with oaths and curses and savage blows, converting into a very hell that home which under other circumstances at the beginning might have been almost a heaven on earth. But it is now too late for any human music to charm such a man, or to tame and "lay" the evil spirit within him.

These things being so, it might be well for city mothers especially to have their daughters take fewer lessons in music, fewer in French, fewer in crochet-work, and more in "common-sense;" more in domestic duties, such as sewing, knitting, patching, darning, dusting rooms, making beds, taking care of their own clothing, and that of the smaller children; helping the mother in all possible ways; thinking for her; planning for her; anticipating her wants and desires and directions; doing all these things, not merely as a duty, but as a pleasure; doing them promptly, cheerfully, and lovingly, at all times and under all circumstances; feeling the while that the child should be the servant, and the mother the served. No one can doubt, that a daughter thus brought up, with frequent opportunities of trying her hand at making cake, baking a loaf, roasting a joint, boiling a potato, drawing a cup of tea, spreading a table, getting up a party, fitting her own dress, trimming her own bonnet, and being her own seamstress, would have a power over a man, all-controlling, in subduing his passions, in chastening his extravagances, and moulding his nature into a form, the very embodiment of all that is noble, manly, generous, and loving.

The "music," then, which the wife should "practice," in order to have a healthful influence over the physical, moral, and mental nature of a man, restraining him from vice, and crime, and gluttony, and late hours, and drunkenness, and the poetry which she should recite to him every day, are the music and poetry of a tidy home, of cleanly and well-behaved children, of quiet and respectful servants, of a table spread so invitingly that if only bread and milk and butter were there. they would taste like nectar and honey just from the hive; while the all-pervading and happy influence of a quiet, loving, and lady-like wife, sanctifies the whole household, and makes it a community of love, of enjoyment, of domestic beatitude.

There must be music and poetry too in the husband; he must strive daily to deport himself toward the woman who has borne him children, with a like respect and deference and consideration and gentleness, to that which he was accustomed to exhibit shortly before the marriage ceremony had made them one. We say "strive," for many a time it will require an effort, a moral power akin to the heroic, for there is much in the life of almost every man of business, so wearying, depressing, and often harrowing to the whole nature, that he would be more than mortal, if under their influences, when the physical nature is tired with labor, he could exhibit the beautiful amenities of an elevated domesticity, without some summoning up to his aid, all the latent power within him, to recall the feelings and affections and deportment of the happy days of courtship. He may sometimes have to contend with woman's waywardness, only exhibited, it may be, when under the influence of sickness, or inward grief, or deep disappointment, or bitter mortification, or of a hard lot in life; but surely it will be the more manly part, under such circumstances, to shut his eye and ear and sense to many things, covering them with that mantle of charity which he should always have at hand, for her sake, who left father and mother and all the dear associations of home and kindred, and threw herself so trustingly on his protection, his love, his honor, and his care. Let the daughter also "practice" for her who bore her, that sweetest of all music to an aged mother's heart, to wit, a prompt, a cheerful, an unhesitating obedience to all her known wishes; let her feel abidingly, that nothing she can do for the mother who loved her and watched over her with so much tenderness and solicitude and anxious care through the running years of infancy and childhood and mature age, can ever half repay her; let that mother's peace and comfort and repose and quiet happiness be the constant study and the steady aim of every dutiful daughter; for however much she may do, it would not be considered half enough when that mother has passed into the grave. Yes, however much she may have done, it will then be felt the strangest thing in the world that she had not done more; she will constantly reproach herself for want of consideration in a thousand little things, each one of which might have been a rill of pleasure to the aged heart as it was nearing its final resting-place.

Let the dutiful and loving daughter "practice" that other "music-lesson" for her mother's sake, *the willingness to learn;* to practice it so diligently, that there need never be a repetition of a mother's counsel, or direction or advice. Said a mother to me once: "I never recollect the time when I found it necessary to repeat a wish to any child of mine; I have only to half tell it when it is done." Happy mother! dear loving children! How I wish there were more such! I know there are too many daughters who are directly the reverse; who seem to think that a mother's advice is out of date; her counsel old fogyish, and all her pains to show her how to do things, are not only disregarded, but are listened to or witnessed with the utmost impatience, as evidenced by the surly look, the unsightly frown, or some disrespectful exclamation. Poor child! every one of these will be a dagger to your heart; the more painful as you grow older; striking deeper and deeper as years roll on, causing many an hour of sadness by day, and of remorses, oh! how grinding! in the sleepless hours of midnight, so many of which are the lot of old age.

The things of which we have been speaking are moral music and moral poetry; these promote the health of the heart; but there are pieces of real, tangible poetry, the repetition or the reading of which aloud, at times, when the mind is in the mellow mood, or when sorrows weigh it down, or when grief presses upon it like a crushing millstone, will many a time lighten the load which burdens poor humanity's heart, and at other times will lift it up, and elevate, and waken it to nobler purposes and to higher resolves, instead of letting life go out in blank despair, or in the dreadful night of suicide.

Poetry and song have not in three thousand years lost any of their efficiency in medicating the maladies of the mind, which, by the way, are sometimes more terrible in their ill-effects, than are physical diseases.

Song soothes the troubled soul; it calms the perturbed spirit, and sweetly lessens the weight of those mournfully pleasing recollections of the far-distant past of childhood and home.

